# Transport and Separation of the Silver Ion with *n*–decanol Liquid Membranes Based on 10–undecylenic Acid, 10–undecen–1–ol and Magnetic Nanoparticles

**DOI:** 10.3390/membranes11120936

**Published:** 2021-11-27

**Authors:** Gheorghe Nechifor, Florentina Mihaela Păncescu, Paul Constantin Albu, Alexandra Raluca Grosu, Ovidiu Oprea, Szidonia-Katalin Tanczos, Constantin Bungău, Vlad-Alexandru Grosu, Mihail-Răzvan Ioan, Aurelia Cristina Nechifor

**Affiliations:** 1 Analytical Chemistry and Environmental Engineering Department, University Politehnica of Bucharest, 1–7 Polizu St., 011061 Bucharest, Romania; ghnechifor@gmail.com (G.N.); florynicorici@yahoo.com (F.M.P.); andra.grosu@upb.ro (A.R.G.); aureliacristinanechifor@gmail.com (A.C.N.); 2 Radioisotopes and Radiation Metrology Department (DRMR), “Horia Hulubei” National Institute for R&D in Physics and Nuclear Engineering (IFIN-HH), 30 Reactorului St., 023465 Măgurele, Romania; razvan.ioan@nipne.ro; 3 Department of Inorganic Chemistry, Physical Chemistry and Electrochemistry, University Politehnica of Bucharest, 1–7 Polizu St., 011061 Bucharest, Romania; ovidiu.oprea@upb.ro; 4 Department of Bioengineering, University Sapientia of Miercurea–Ciuc, Libertatii St., 500104 Miercurea–Ciuc, Romania; tczszidonia@yahoo.com; 5 Department of Engineering and Management, Faculty of Management and Technological Engineering, University of Oradea, 410087 Oradea, Romania; bungau@uoradea.ro; 6 Department of Electronic Technology and Reliability, Faculty of Electronics, Telecommunications and Information Technology, University Politehnica of Bucharest, Iuliu Maniu Blvd., nr. 1–3, 061071 Bucharest, Romania

**Keywords:** bulk liquid membranes, 10-undecilenic acid carrier, 10-undecenol carrier, silver separation, silver transport, magnetic nanoparticles, oxide nanoparticles, turbulence promotors

## Abstract

This paper presents a transport and recovery of silver ions through bulk liquid membranes based on *n*–decanol using as carriers 10–undecylenic acid and 10–undecylenyl alcohol. The transport of silver ions across membranes has been studied in the presence of two types of magnetic oxide nanoparticles obtained by the electrochemical method with iron electrodes in the electrolyte with and without silver ions, which act as promoters of turbulence in the membrane. Separation of silver ions by bulk liquid membranes using 10–undecylenic acid and 10–undecylenyl alcohol as carriers were performed by comparison with lead ions. The configuration of the separation module has been specially designed for the chosen separation process. Convective-generating magnetic nanoparticles were characterized in terms of the morphological and structural points of view: scanning electron microscopy (SEM), high-resolution SEM (HR–SEM), energy dispersive spectroscopy analysis (EDAX), Fourier Transform InfraRed (FTIR) spectroscopy, thermal gravimetric analysis (TGA), differential scanning calorimetry and magnetization. The process performance (flux and selectivity) was tested were tested for silver ion transport and separation through *n*–decanol liquid membranes with selected carriers. Under the conditions of the optimized experimental results (pH = 7 of the source phase, pH = 1 of the receiving phase, flow rate of 30 mL/min for the source phase and 9 mL/min for the receiving phase, 150 rot/min agitation of magnetic nanoparticles) separation efficiencies of silver ions of over 90% were obtained for the transport of undecenoic acid and about 80% for undecylenyl alcohol.

## 1. Introduction

The liquid membrane consists of a distinct organic phase that separates two other aqueous phases [1]. The aqueous phase containing the species about to be transported is called the source phase (SP) and the aqueous phase that receives the transported species is called the receiving phase (RP) [2]. Membrane processes are individualized through the use of the ion carrier, the carrier (C) that exists in the organic phase (the membrane) compared to the traditional extraction system, which uses complexants. Proper selection of the carrier is crucial for the efficiency of liquid membranes. High separation selectivity factors are achieved when the chosen carrier has a high affinity for one of the components of the feed solution [2,3].

The main advantage of liquid membranes is that the solubility and diffusion coefficients of the compounds in a liquid medium are higher than in a solid medium, and the addition of a transport agent further grows the permeability of the membrane [4,5].

**Figure 1:**
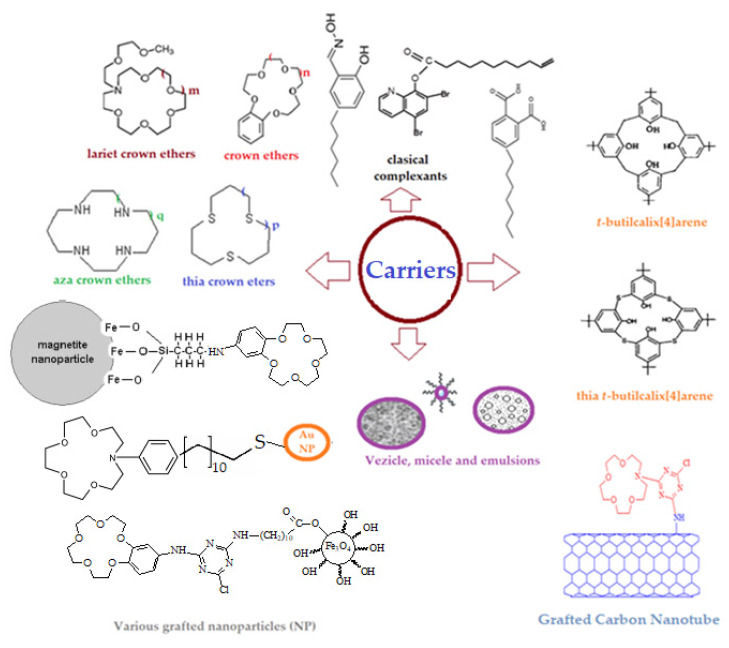
Usual types of carriers: macrocyclic compounds, modified classical complexants, nanospecies (m, n, p and q = −1, 0, 1, 2).

The pure membrane solvent rarely ensures the selectivity of the separation process, that is why specific complexants or carriers are introduced in the liquid membrane (Figure 1): macrocyclic compounds (crown ethers, cryptans, spherants, calixarenes, cyclodextrins) [6,7], chemically modified classical complexants (especially with groups lipophilic) [8], functionalized nanospecies (nanoparticles, nanotubes, vesicles, micelles) [9,10,11].

The main advantages of liquid membranes are high selectivity, guided by the use of adequate carriers, accessibility of laboratory experiments, but also on a pilot or industrial scale [12,13], diversity of technical manufacturing methods: bulk liquid membranes (BLM), liquid membranes on support (SLM), or emulsion liquid membranes (ELM) [14,15,16,17].

Other advantages of the liquid membranes: they are simple in concept and operation, are modular and easy to achieve on a large scale, have low energy consumption, and therefore, have a remarkable potential for impact on the environment and energy issues [18,19,20].

Thus, the liquid membranes continue to be a viable alternative both for classical separation processes (precipitation, ion exchange, adsorption, extraction) and the other membrane methods (polymers, composites, ion exchangers) [19,20,21,22].

However, the liquid membranes also have important disadvantages that current research is trying to either reduce or overcome [23,24,25,26]. Basically, the problems to be solved are related to finding new carriers (to increase and direct the selectivity), increasing the mass transfer surface (adapting the design of each type of membrane), improving the membrane stability and avoiding solvent losses (including the use of green solvents), achieving convective transport in and through the membrane [27,28,29,30,31].

Reducing the number of chemical substances in the process of separation with liquid membranes is also a priority that can place them among the ecological processes and also increase their competitiveness with other types of membranes [32,33].

Bulk liquid membranes remain the basic tools in the laboratory study of new carrier performances. At the same time, in these cases, the design of the permeator must be adapted to the requirements of the considered separation and transport process, and this objective is easily feasible in the case of bulk liquid membrane permeates. This study aims at introducing in the practice of membrane separations a bifunctional, accessible, biodegradable carrier of known biomedical interest, namely 10–undecylenic acid (undecenoic acid) [34,35,36,37,38,39,40,41], as well as testing a new type of permeation module in the process of transport and separation of silver ions.

This paper addresses the use of undecylenic acid and 10–undecylenyl alcohol as carriers, for the transport and separation of silver ions in a bulk liquid membrane system based on *n*-decanol, with magnetic convection promoters based on oxide nanoparticles.

## 2. Materials and Methods

### 2.1. Reagents and Materials

The reagents and materials used in the presented work were of analytical grade.

They were purchased from Merck (Merck KGaA, Darmstadt, Germany): iron (wire), silver nitrate, lead nitrate, sodium chloride, sodium hydroxide, hydrochloric acid, nitric acid; and, from Sigma-Aldrich (Merck KGaA, Darmstadt, Germany): 10–undecylenic acid (undecenoic acid, molar mass: 184.28 g/mol, density: 912 kg/m^3^, solubility in water: 0.074 mg/mL), undecylenyl alcohol (10–undecen–1–ol, or 11–hydroxy–1–undecene, molar mass: 170.29 g/mol, density: 846 kg/m^3^, solubility in water: 0.061 g/L), *n*–decyl alcohol (molar mass: 158.28 g/mol, density: 830 kg/m^3^, solubility in water: 0.037 mg/L).

The purified water characterized by 18.2 μS/cm conductivity was obtained with a RO Millipore system (MilliQ^®^ Direct 8 RO Water Purification System, Merck, Darmstad, Germany). The tubular dialysis membranes were from Visking (Medicell Membranes Ltd., London, UK).

### 2.2. Methods

#### 2.2.1. Preparation of the Oxide Magnetic Nanoparticles

The method of obtaining iron-based magnetic nanoparticles is the electrochemical one, previously presented in detail [42,43]. In this case, the electrolysis is performed in pure water (to obtain magnetic nanoparticles of iron oxides) and in silver nitrogen electrolyte 10^−1^ mol/L (to obtain magnetic nanoparticles based on silver and iron oxides).

In this paper, the nanoparticles are obtained using silver nitrate as an electrolyte, in order to avoid impurity with chloride anions which were found in the nanoparticles obtained previously.

A 250 mL volume of the considered solution (pure water or silver nitrate) was introduced into an electrolysis cell of the PARSTAT 2273 Potentiostat (Princeton Applied Research, AMETEK Inc., Oak Ridge, TN, USA). This equipment is provided with three electrodes: pure iron anode and cathode, and a platinum wire as a reference. Cyclic voltammetry was performed for a potential sweep between −0.5 and +1.23 V at a scan rate of 50 mV/s. The experimental procedure took place at room temperature [42].

After six hours of work, black nanoparticles were collected at the cell base. Afterward, they were magnetically transferred into a dialyzer with a regenerated cellulose membrane. Continuous dialysis was performed until a pH sample was obtained. The pH was followed up with a combined selective glass electrode (HI 4107, Hanna Instruments Ltd., Leighton Buzzard, UK) and a multi-parameter system (HI 5522, Hanna Instruments Ltd., Leighton Buzzard, UK) [43].

The samples of nanoparticles containing iron oxide or iron-silver oxide were characterized by scanning electron microscopy (SEM and HFSEM), EDAX, magnetization, UV-Vis and atomic absorption spectroscopy, and thermal analysis (TG, DSC) [44].

#### 2.2.2. Obtaining the Liquid Membranes on *n*–decyl Alcohol–Oxide Nanoparticles

A volume of 600 mL (about 498 g), *n*-alcohol (*n*-decanol), and 2.0 g of iron or silver-iron oxide nanoparticles were placed in an 800 mL glass tank. In this system, 1 g of 10–undecen–1–ol is then added. In another 800 mL vessel, the same components are introduced and then 1 g of 10–undecylenic acid is added.

Every glass tank was placed in an ultrasonic bath (Elmasonic S, Elma Schmidbauer GmbH, Singen, Germany) for two hours while observing the complete dispersion. Finally, a dark brown dispersed liquid system was formed.

#### 2.2.3. The Transport and Separation of the Silver Ions

**Figure 2:**
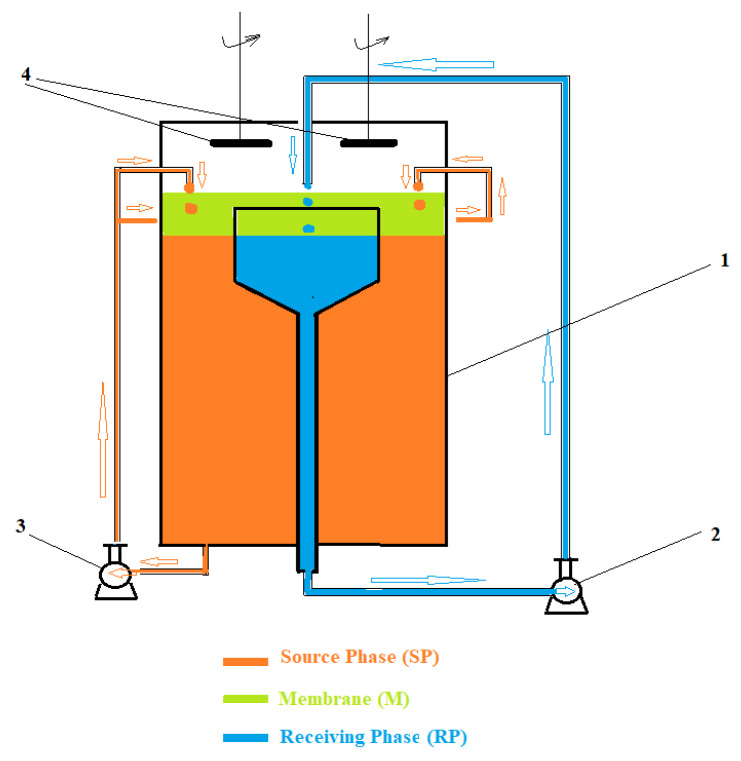
The schematic presentation of the bulk liquid membrane module: 1—the body of the module; 2 and 3: peristaltic pumps; 4—agitators with magnetite rod.

The study of transport and separation was performed in an installation, with a permeation module (cell) (1), made using simple laboratory means (Figure 2): a 1000 mL cylindrical glass vessel, a conical–cylindrical glass funnel (with a useful volume of 150 mL) and silicone rubber connecting tubes. The circulation of the fluids that constitute the source phase (SP) and receiving phase (RP) is performed using peristaltic pumps (2,3) and the agitation of the membrane particles with magnetite wand shakers (4).

The laboratory installation allows the variation of the source and receiving phase flows, and the stirring speed of magnetic rods (rotations/min).

The monitoring of the concentration of chemical species is performed directly in the system (with selective ion microsensors). The validation is done by periodic sampling (every 30 min) and spectrometric analyses (UV-Vis and atomic absorption).

The flows of the silver ions (or lead ions) from the source phase [45,46] were determined against the measured permeate mass within a determined time range, applying the following formula:

(1) $$J=\frac{M}{S\cdot t}\;(\mathrm{mg}/(m^{2}\;s))\;\mathrm{or}\;((\mathrm{mol})/(m^{2}\;s))$$

*M* being the permeate mass (g or mol), *S* being the effective surface of the membrane (m^2^), and *t* the time (s) necessary to collect the permeate volume.

The extraction efficiency (EE %) for the species of interest using the concentration of the solutions [33,34] was calculated as follows:

(2) $$EE\left(\%\right)=\frac{\left(c_{0}-c_{f}\right)}{c_{0}}\cdot 100$$

*c_f_* being the final concentration of the solute (considered chemical species) and *c*_0_ the initial concentration of solute (considered chemical species).

The same extraction efficiency can also be computed based upon the absorbance of the solutions [47,48,49,50], as in:

(3) $$EE\left(\%\right)=\frac{\left(A_{0}-A_{s}\right)}{A_{0}}\cdot 100$$

*A*_0_ being the initial absorbance of the sample solution and *A_s_* the current absorbance of the sample.

### 2.3. Equipment

The microscopy studies, SEM and HFSEM, were performed on a Hitachi S4500 system (Hitachi High-Technologies Europe GmbH, Krefeld, Germany).

Thermal analysis, TG-DSC, was performed with a STA 449C Jupiter apparatus, from Netzsch (NETZSCH-Gerätebau GmbH, Selb, Germany). Each sample weighed approximately 10 mg. The samples were placed in an open alumina crucible and heated up to 900 °C, at a 10 K∙min^−1^ rate, under a flow of 50 mL∙min^−1^ dried air. As a reference, we used an empty alumina crucible. The evolved gases were analyzed with an FTIR Tensor 27 from Bruker (Bruker Co., Ettlingen, Germany), equipped with a thermostatic gas cell.

The nanoparticle magnetization diagrams were determined with Quantum Design MPMS 3 Magnetometer (Quantum Design Europe, Darmstadt, Germany) based on superconducting quantum interference device detection (SQUID). The DC operation mode applied allowed to run SQUID -vibrating- sample magnetometer (VSM) measurements.

To assess and validate the content in metal ions, the atomic absorption spectrometer AAnalyst 400 AA Spectrometer (Perkin Elmer Inc., Waltham, MA, USA) with WinLab32—AA software (Perkin Elmer Inc., Waltham, MA, USA), with a single–element hollow–cathode lamp was used. The operating current was set up at 2 mA, wavelength 248.3 nm, and 0.2 nm spectral bandwidth for determining the iron content. For silver, the values of the experimental parameters are a wavelength of 328.1 nm, and a 0.7 nm spectral bandwidth, at an operating current of 5 mA.

The UV–Vis studies on the nanoparticles samples were performed on dual-beam UV equipment–Varian Cary 50 (Agilent Technologies Inc., Santa Clara, CA, USA) at a resolution of 1 nm, spectral bandwidth 1.5 nm, and 300 nm/s scan rate. The UV-Vis spectra of the samples were recorded for a wavelength from 200 to 800 nm, at room temperature, using 10 mm quartz cells.

Spectroscopy Bruker Tensor 27 Fourier Transform Infra-Red (FTIR) with Diamond ATR (Bruker Optik GmbH, Ettlingen, Germany) was used to study the interactions between the chemicals used in the developed membranes. FTIR analysis was recorded in the range of 500 to 4000 cm^−1^.

The UV–Vis analysis of the silver ions solutions was performed on a CamSpec M550 spectrometer (Spectronic CamSpec Ltd., Leeds, UK).

The UV–Vis spectra of the samples were recorded for a wavelength ranging from 200 to 800 nm, at room temperature, using 10 mm quartz cells.

The electrochemical analysis was followed up with a PARSTAT 2273 Potentiostat (Princeton Applied Research, AMETEK Inc., Oak Ridge, TN, USA). A setup with a glass cell with three electrodes has been used.

The chloride anion concentration (in receiving phase) was determined using a combined selective chloride electrode (HI 4107, Hanna Instruments Ltd., Leighton Buzzard, UK) and a multi-parameter system (HI 5522, Hanna Instruments Ltd., Leighton Buzzard, UK).

## 3. Results and Discussions

In terms of applications in chemical synthesis, undecylenic acid is an excellent spacer and surface coating agent: nanoparticles, nanotubes, films, and membranes [51,52,53,54].

The use of undecylenic acid as a carrier in liquid membranes has several arguments that derive from its bifunctional chemical structure: surface agent properties, the ability of the carboxyl group to interact with various metal ions, the specific tendency of the double bond (C=C) to interact with silver, high solubility in alcohols and insolubility in water.

**Figure 3:**
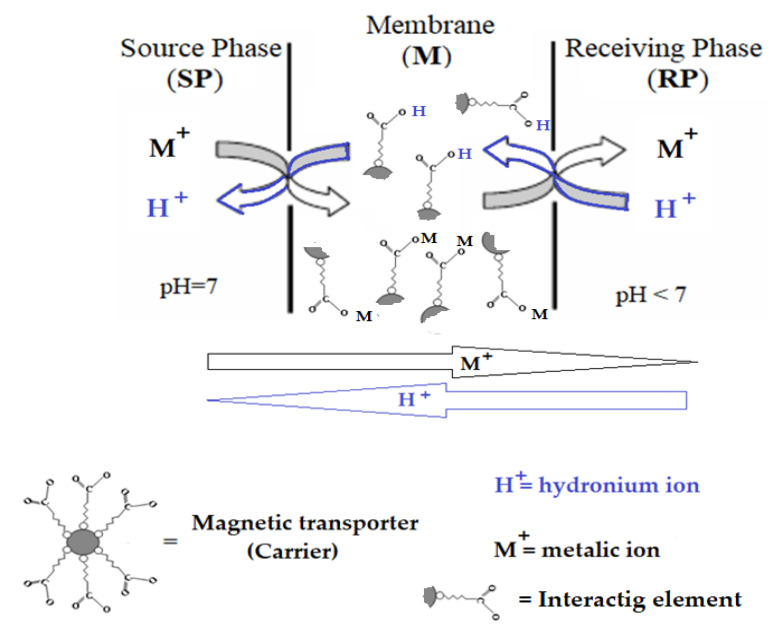
Membrane system considered for the separation of silver ion with carrier undecenoic acid and magnetic oxide nanoparticles.

To highlight the transport characteristics, a coupled transport type system with a carrier was chosen (Figure 3). The membrane consists of *n*–decanol in which magnetic oxide nanoparticles coated with undecenoic acid are dispersed. The source phase (SP) consists of a silver nitrate and silver nitrate and/or lead nitrate solution in equimolar concentrations, dissolved in ultrapure water. The receptor phase (RP) consists of a solution of hydrochloric acid, with a concentration between 10^−5^ mol/L and 10^−1^ mol/L.

To highlight the influence of the carboxyl group and implicitly of the alkylene group, all experiments were duplicated using 10–undecen–1–ol as a control carrier.

The use of the permeator from Figure 2 allows the variation of the following parameters: the volume of membrane phases, the flow rate of the aqueous phases in the system (source phase and receiving phase), the rotation speed of the magnetite rods that drive the turbo-slow promoters (magnetic oxide nanoparticles), the nature of the magnetic particles in the membrane phase, the nature of membrane carriers, the composition of aqueous phases (pH of the source and receptor phase).

The experimental study performed has three main objectives, each with specific activities: preparation and characterization of oxide magnetic nanoparticles, transport of silver ions through bulk liquid membranes with undecenoic acid carrier and magnetic nanoparticles, and separation of silver ions from lead ions by bulk liquid membranes using undecylenyl alcohol and undecenoic acid as carriers.

### 3.1. The Preparation and Characterization of Magnetic Nanoparticles

To obtain the iron oxide magnetic nanoparticles in the electrolytic silver nitrate solution, cyclic voltammetry with pure iron electrodes and a platinum reference electrode was chosen, and the potential was swept between −0.5 and +1.23 V.

The following reaction steps are known for the electrolysis mechanism in electrolyte without interfering electroactive species [55,56,57,58,59]:

Fe ⇄ Fe^2+^ + 2e^−^ (4)

Fe^2+^ ⇄ Fe^3+^ + 1e^−^ (5)

H_2_O ⇄ 2H^+^ + 2e^−^ + ½O_2_ (6)

2H_2_O + 2e^−^ ⇄ H_2_ + 2OH^−^ (7)

Fe^3+^ + OH^−^ ⇄ Fe(OH)_3_(s) (8)

3Fe(OH)_3_(s) + H^+^ + e^−^ ⇄ Fe_3_O_4_(s) + 5H_2_O (9)

The silver discharge potential, which is affected by the equilibrium constants of the reactions, imposes the electrolysis potential to which the silver ion aqueous complexes, used as electrolytes, are discharged [50].

In the study, magnetic nanoparticles were obtained by electrolysis in pure water (NP–Fe) and in silver nitrate solution (NP–Fe–Ag).

**Figure 4:**
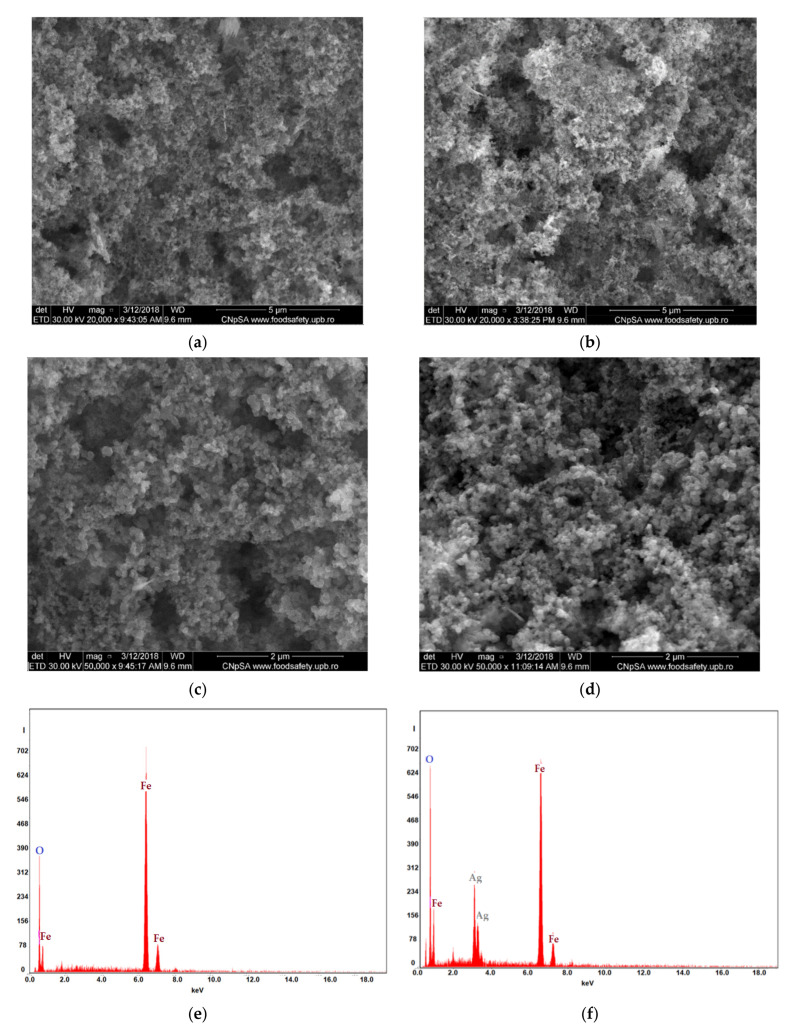
Characteristics of iron-based oxide nanoparticles (NP–Fe) (**a**,**c**,**e**); and iron and silver (NP–Fe–Ag) (**b**,**d**,**f**): scanning electron microscopy (SEM) (**a**–**d**); and energy dispersive spectroscopy analysis (EDAX) (**e**,**f**).

**Figure 5:**
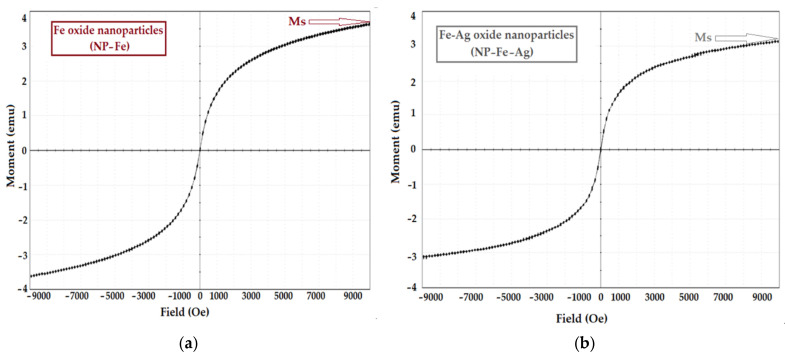
The characteristics of magnetic nanoparticles rendered by the magnetization curves of oxide nanoparticles: iron (**a**); and iron and silver (**b**) (Ms–magnetization of saturation).

The main characteristics of the obtained nanoparticles are given in Figure 4, which presents the results of the examination by scanning electron microscopy (SEM) and energy dispersive spectroscopy analysis (EDAX), and in Figure 5, which shows the magnetization curves.

Oxide nanoparticles have a similar morphology for both types of nanoparticles (Figure 4a–d). The nanoparticle sizes range between 20 and 50 nm, detailed for a wider range of electrolytes [42,43,55,56,57].

The surface composition of the nanoparticles reveals Fe and O in the case of nanoparticles obtained by electrolysis in pure water, and Fe, O and small amounts of Ag for the nanoparticles obtained by electrolysis in silver nitrate solution (Figure 4e,f).

The saturation magnetization of iron oxide-based nanoparticles (NP–Fe) is slightly higher than that of iron and silver oxide nanoparticles (NP–Fe–Ag) (Figure 5a,b).

**Figure 6:**
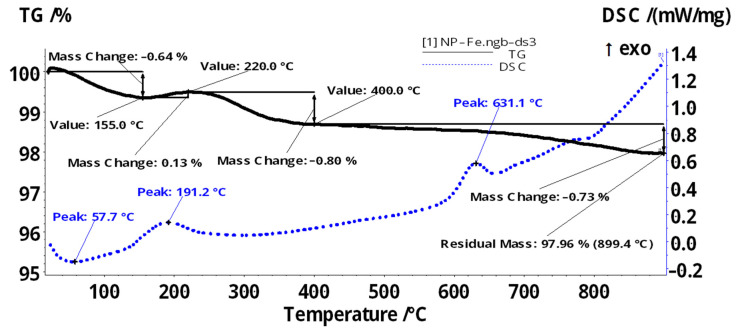
Thermal characteristics of iron oxides based magnetic nanoparticles (NP–Fe).

**Figure 7:**
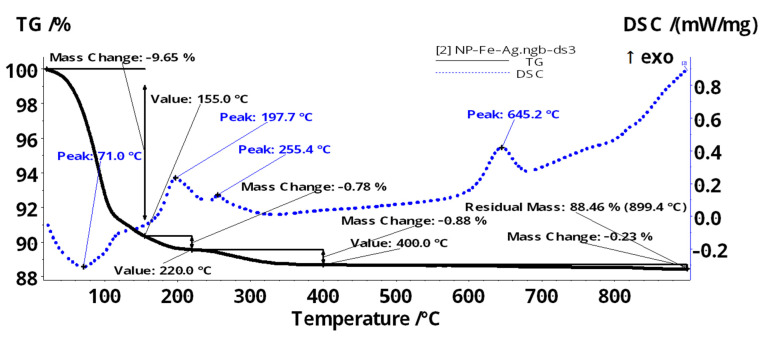
Thermal characteristics of iron oxides based magnetic nanoparticles (NP–Fe–Ag).

**Figure 8:**
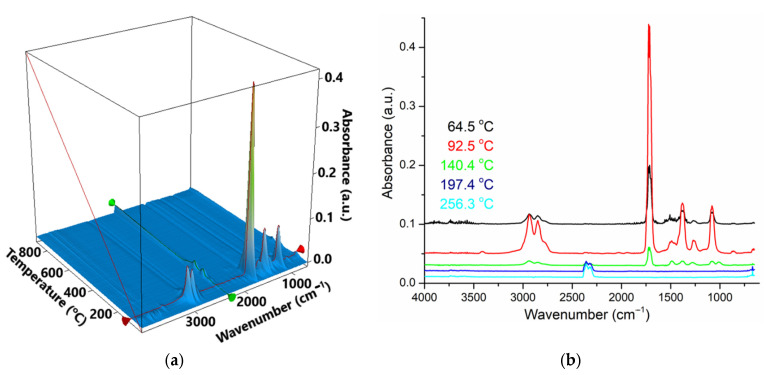
The 3D plot of FTIR spectrum vs. temperature for the evolved gases from the NP–Fe–Ag sample (**a**); Individual FTIR spectra for the evolved gases in NP–Fe–Ag sample, at 65, 93, 140, 197 and 256 °C (**b**).

The thermal gravimetric analysis, differential scanning calorimetry (TGA), and Fourier Transform InfraRed (FTIR) spectroscopy for the evolved gases (Figure 6, Figure 7 and Figure 8) complete data regarding the nanoparticles dispersed in *n*-decanol in the presence of 10–undecylenyl alcohol and undecenoic acid.

The working technique, thermal analyzer coupled with the Fourier transform infrared spectrometer (FTIR), allows the highlighting of chemical species desorbed and thermally degraded depending on the temperature.

For iron oxide based magnetic nanoparticles (NP–Fe and NP–Fe–Ag) the specific thermal diagrams are presented in Figure 6 and Figure 7. These thermal diagrams were obtained for the particles recovered from the membrane dispersion, in order to observe the interaction with the carrier and/or the working solvent.

The iron oxide nanoparticles (NP–Fe) sample (Figure 6) is losing 0.64% of the initial mass in the temperature interval RT–155 °C. This process is accompanied by an endothermic effect, with a minimum at 57.7 °C. It can be attributed to the elimination of some solvent molecules (water) adsorbed on the surface of the nanoparticles. After 155 °C the sample mass is slightly increasing, with 0.13%, up to 220 °C, in an exothermic process. This can be attributed both to the oxidation of Fe (II) to Fe (III) and to the transformation of magnetite to maghemite. Between 220–400 °C the sample is losing 0.80% of initial mass, most probably by eliminating the surface –OH moieties. The exothermic effect from 631.1 °C is attributed to the phase transition magnetite to hematite, a characteristic effect for such samples [60]. The residual mass is formed by red Fe_2_O_3_, representing 97.96%.

The sample of iron-silver oxide nanoparticles (NP–Fe–Ag) (Figure 7) is losing 9.65% of the initial mass between RT–155 °C, the process being accompanied by an endothermic effect, with the minimum at 71 °C. This can be attributed to the elimination of the adsorbed solvent molecules–the vibrations around 2900–3000 cm^−1^ (characteristic to C_sp3_–H^–^) can be seen in the FTIR 3D plot (Figure 8a).

The sample continues to lose mass (0.78% up to 220 °C and 0.88% up to 400 °C), the elimination of the solvent and of surface –OH moieties being overlapped with the oxidation of magnetite to maghemite (the exothermic effect from 197.7 °C). The phase transition from magnetite to hematite occurs at 645.2 °C, as indicated by the exothermic effect on the DSC curve. The residual mass is 88.46% and is composed of red Fe_2_O_3_.

The most important peaks are at 2939 and 2850 cm^−1^ (C_sp3_–H)—for the organics in evolved gases; 2355 and 2322 cm^−1^ for CO_2_ at temperatures over 200 °C; 1724 and 1714 cm^−1^ for the most intense band (could be C=O); 1488, 1381, 1278, 1081 and 1009 cm^−1^ are in the fingerprint area (Figure 8b).

At low temperatures (Figure 8a,b) some organics are constituted of the major components of the evolved gases. At 197 °C and 256 °C, there are two small evolving events, in which CO_2_ is the identified component (can be due to burning the organic molecules still present on nanoparticle surface).

The silver deposited on the surface of magnetic iron oxide nanoparticles can be oxidized at low temperatures, but only if it is not protected by an organic layer (which is the case in this sample). The removal of the organic adsorbed molecules in terms of mass loss and endothermic effect would mask such a process (the oxidation of silver). If the silver is deposited in Ag_2_O form, then the oxide is decomposed in the interval 150–200 °C. This process is overlapped with oxidation of Fe^2+^ to Fe^3+^ and therefore, given the low silver content, we can not detect such decomposition. At higher temperatures, silver is inert and no other reactions can be identified for it.

The thermal study shows that nanoparticles containing silver (NP–Fe–Ag) adsorb a detectable amount of organic compounds (solvent and/or carriers).

### 3.2. The Transport of Silver Ions through Bulk Liquid Membranes with Undecenoic Acid and Magnetic Nanoparticles Carrier

**Figure 9:**
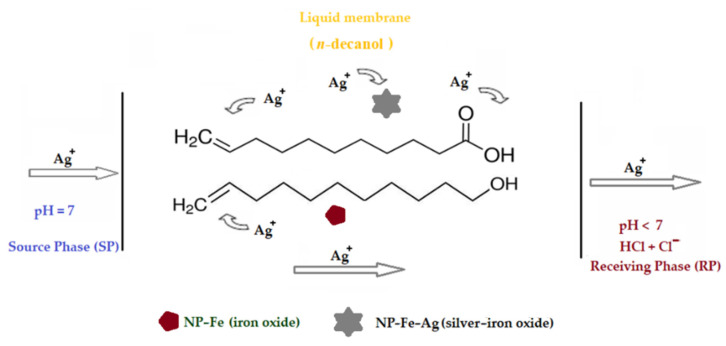
Possible interactions carrier–silver ions when using *n*–decanol with carriers: undecenoic acid, 10–undecylenyl alcohol and magnetic nanoparticles based membranes.

The silver ion is very often found in the separation, concentration and purification with liquid membranes due to the need for its recuperative separation, but also for its well-known biocidal effect [61,62,63,64]. In the particular case of this study, the silver ion is an important tracer because it has a specific interaction with undecenoic acid through the double bond, but also a nonspecific one due to the carboxyl group. Transport tests have a theoretical importance, regarding the introduction of an accessible bifunctional compound as a carrier in liquid membranes, but also an applicative importance, considering the biomedical importance of both the carriers and the silver ion. Testing of 10–undecylenyl alcohol as a carrier was performed under the same experimental conditions in order to highlight the effect of the carboxyl group for the considered transport and, implicitly, the contribution of the alkylene group (double bond) in this process (Figure 9).

**Figure 10:**
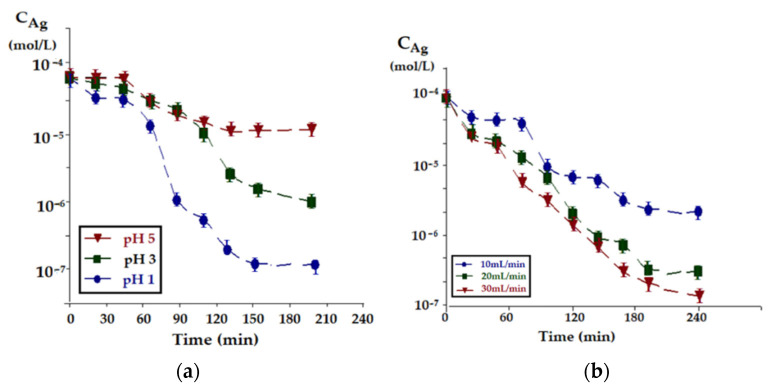

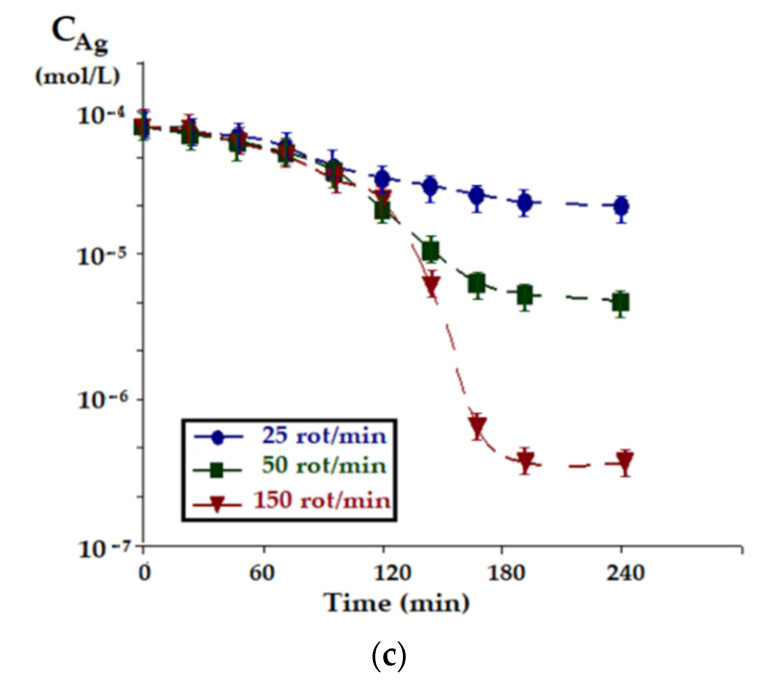
The evolution of silver ions concentration in the source phase: (**a**) depending on the pH of the receiving phase, (**b**) depending on its flow, (**c**) as a function of magnetic nanoparticles stirring speed in the membrane.

The experiments of transporting the silver ion through bulk liquid membranes based on *n*–decanol, containing as a carrier the undecenoic acid, followed the influence of the pH of the receptor phase, the role of the carrier and oxide magnetic nanoparticles in the membrane system, the flow effect of the two aqueous phases, source and receptors and the influence of magnetic agitation of the liquid membrane containing magnetic oxide nanoparticles (Figure 10).

In the experiments performed, the concentration of nanoparticles and carriers was the same, following the hydrodynamic parameters of the liquid membrane and the receiving phase on the transport of silver ions.

In the receiving phase, the concentration of chloride ions was kept relatively constant using a solution of sodium chloride of concentration 2.0 mol/L, and the pH was imposed by the concentration of hydrochloric acid (10^−5^ mol/L up to 10^−1^ mol/L) so that the pH gradient is a support of the driving force of the transfer of silver ions. The source phase consists of a solution of silver nitrate in ultra-pure water with a concentration of 10^−4^ mol/L.

Figure 10a shows the evolution of silver ions concentration in the source phase depending on the pH of the receiving phase. Hydrodynamic parameters (source phase flow, receiving phase flow and magnetic agitation of membrane nanoparticles) was constant but not optimized. Increasing the pH gradient, the pH of the receiving phase compared to the pH = 7 of the source phase, leads to an increase in the transport speed over the entire studied range.

The insignificant increase variations of silver ions concentration in the source phase towards the end of the working time (at pH = 1 in the receiving phase) has multiple explanations related to the error induced by the cotransport of the protons from the receiving phase to the source phase and also to the slight measuring errors at high dilutions. After approximately 60 min of operation, there is an acceleration of the transport speed for all studied systems.

In the case of the experiments aiming at the influence of the recirculation of source phase flow (Figure 10b), an acceleration of the transport speed is found after ~80 min of operation, for all the studied systems. The transport speed increases over the entire operating interval, as the flow increases.

For imposed conditions regarding the silver ion concentration in the source phase at pH = 1, constant flow (5 mL/min) of the receiving phase and a flow of 20 mL/min for the source phase, silver ion transport depending on the stirring speed of magnetic nanoparticles in the membrane (Figure 10c), this concentration increases with higher rotational speeds of the magnetic rods that generate the membrane convection. The effect of magnetic convection is more pronounced after about 120 min of operation.

The relative level in the first part of the process shows that the flow of matter is relatively constant during the process, which allows a rapid evolution when the supply concentration decreases.

**Figure 11:**
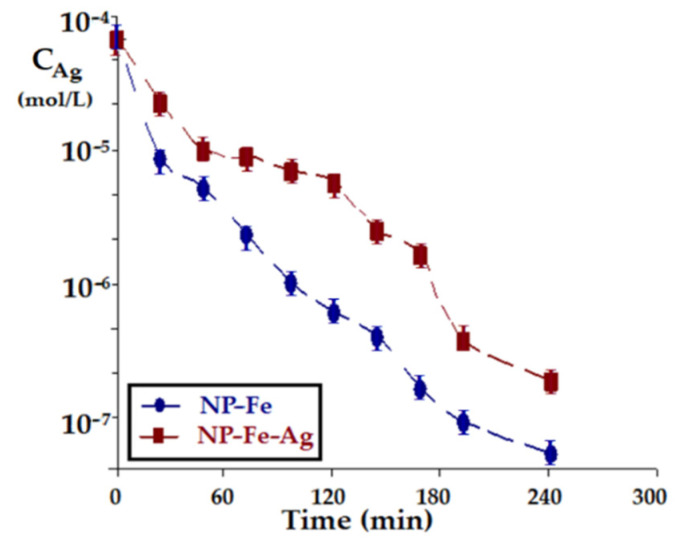
Evolution of silver ion concentration in the source phase depending on the nature of the nanoparticles, at a flow rate of 30 mL/min in the source phase with pH = 1; and constant flow (5 mL/min) of the receiving phase, with magnetic stirring of nanoparticles in the membrane (150 rot/min).

The evolution of the transport of silver ions from the source phase, at the flow of 30 mL/min, the source phase at pH = 1 and constant flow (5 mL/min) of the receiving phase, with the magnetic agitation of the nanoparticles in the membrane (150 rot/min), depending on the nature of the nanoparticles (Figure 11) shows a constant increase over the entire time interval studied, being more accentuated for the iron oxide nanoparticles than for the iron and silver oxide nanoparticles.

The blocking of the double bond in the carrier by interaction with the silver ions from the NP–Fe–Ag nanoparticle, leads to the diminution of the possibilities of taking over the silver ions from the source phase. Basically, the carrier is free to interact with the silver ions in the source phase with both the carboxyl group and the double bond, in the case of the NP–Fe nanoparticle, while in the case of the NP–Fe–Ag nanoparticle the alkenic group is largely unavailable to transport, being in contact with the nanoparticle, the transport being thus ensured only by the carboxyl group.

**Figure 12:**
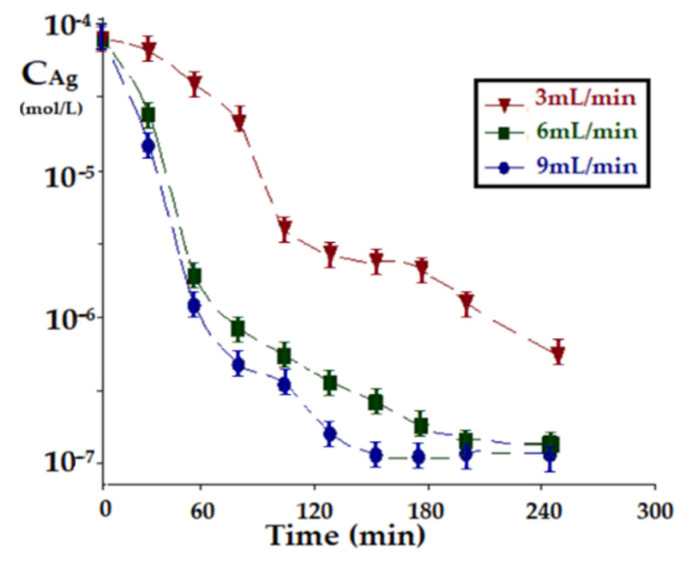
The evolution of silver ions concentration in the source phase depending on the flow of the receiving phase, at the flow of 30 mL/min of the source phase and pH = 1 and with the magnetic agitation of the nanoparticles in the membrane (150 rot/min), for iron oxide nanoparticles.

The evolution of the silver ion concentration at the flow rate of 30 mL/min of the source phase with pH = 1 and with the magnetic agitation of the nanoparticles in the membrane (150 rot/min), for iron oxide nanoparticles in the source phase depending on the flow of the receiving phase (Figure 12) shows a marked increase over the entire working period. Unlike previous experiments, in this case, the initial period of relative stagnation is missing, especially at higher flow rates of the receiving phase.

The study presented in this section reveals that the rate of silver ion transport in a liquid membrane permeator based on *n*–decanol, containing oxide magnetic nanoparticles and undecenoic acid carrier depends on the flow of the source and receiving phase, magnetic agitation of oxide nanoparticles in the membrane, pH of the receiving phase, the nature of the magnetic particles in the liquid membrane.

For the permeator presented in Figure 2, using *n*–decanol as the liquid membrane and undecenoic acid carrier, the following values lead to the best results of silver ion transport, source phase flow: 30 mL/min, receiving phase flow: 9 mL/min, magnetic stirring of oxide nanoparticles in the membrane: 150 rot/min, pH of the receiving phase: 1, and the nature of magnetic particles in the liquid membrane: iron oxide.

**Figure 13:**
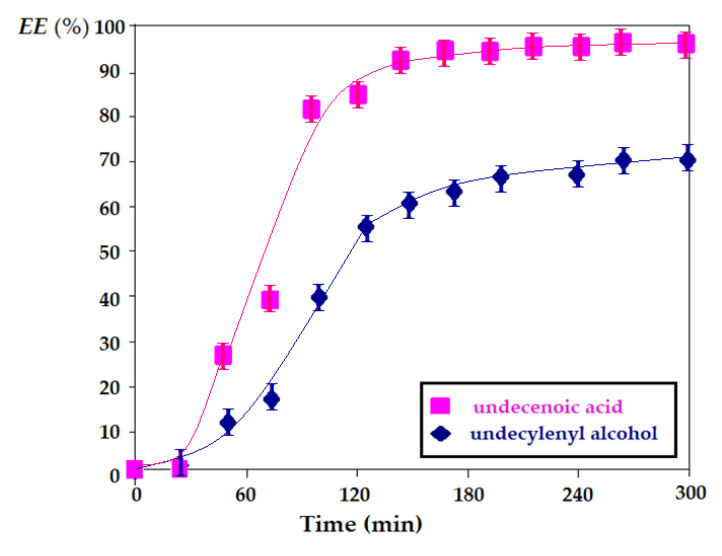
Silver ion extraction efficiency for undecenoic acid compared to undecylenyl alcohol under similar operating conditions.

Under these conditions, the extraction efficiency (EE) was determined for undecenoic acid compared to undecylenyl alcohol (Figure 13).

The obtained results show that undecenoic acid is much more efficient in separating and transporting the silver ion than undecylenyl alcohol, revealing the superior contribution of the carboxyl group to the alcoholic one, but also the definite interaction of the silver ion with the alkene double bond.

### 3.3. Separation of Silver Ions from Lead Ions by Bulk Liquid Membranes with Undecylenyl Alcohol and Undecenoic Acid Carriers

The experiments performed for the transport and separation of silver and lead cations from an equimolar solution with pH = 7 in the source phase and pH = 1 in the receiving phase were carried out based on the operational parameters previously established: source phase flow: 30 mL/min, receiving phase flow: 9 mL/min, and magnetic stirring of oxide nanoparticles from the membrane: 150 rot/min, using turbulence generating particles in the membrane, based on *n*–decanol and having as carriers, either undecylenyl alcohol, or undecenoic acid.

**Figure 14:**
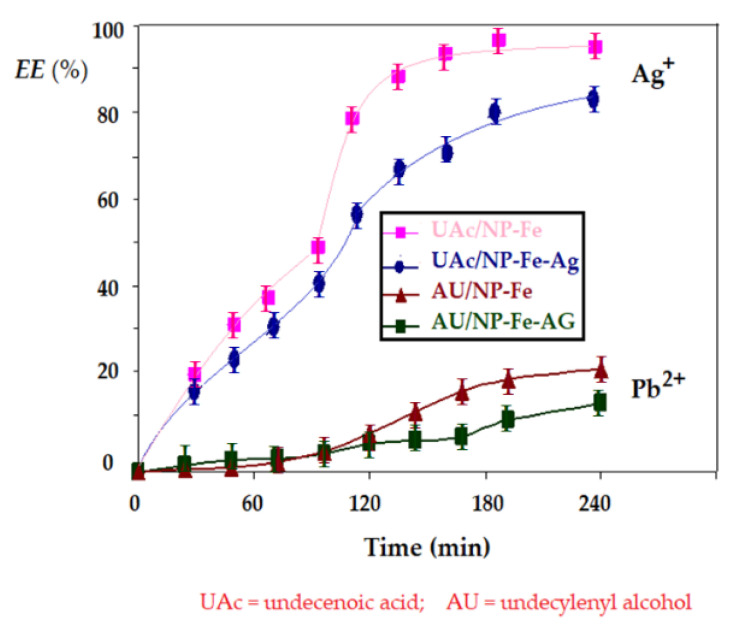
Efficiency of silver and lead ion extraction from the solution in equimolar concentration (10^−4^ mol/L), with pH = 7, at a flow rate of 30 mL/min, for undecenoic acid, compared to undecylenyl alcohol, at a flow rate of 9 mL/min and pH = 1 in the source phase.

The results of the experiments are shown in Figure 14. The extraction efficiency of silver and lead ions differs, under the established working conditions, being clearly superior in the case of silver. There are also noticeable differences in the results of the comparative efficiency of the two carriers and, respectively, the oxide nanoparticles used. Thus, for transport with undecenoic acid, better results are obtained compared to undecylenyl alcohol, with the ferric oxide nanoparticles having a better extraction efficiency than iron and silver oxide nanoparticles. The selectivity factor for silver ions compared to lead ions exceeds the value of six units in all cases studied, indicating the possibility of separation of the two ions. It is very interesting to note that, although the extraction efficiency in the case of undecylenyl alcohol is lower, the separation factor is higher (it reaches eight units). These results indicate that the silver ion is transported simultaneously by interaction with the carboxyl and alkylene groups–for undecenoic acid, and by interaction with the double alkylene bond in the case of undecylenyl alcohol. At the same time, in the case of lead ions, for undecenoic acid, the transport is given by the interaction with the carboxyl group. For undecylenyl alcohol, the transport mechanism must be approached through additional research, adsorptions on nanoparticles, or embedding in micelles.

The experimental study undertaken for the separation of silver and lead ions led to the validation of the interaction hypotheses illustrated in Figure 9:

- The silver ion interacts with both the carboxyl and the alkylene type group in undecenoic acid,
- The carboxyl groups of undecenoic acid interact with iron and silver ions from iron oxide based nanoparticles,
- The alkylene group interacts with the silver ions in the silver and iron oxide nanoparticles,
- Undecylenyl alcohol has an interaction with the silver ions in the silver and iron oxide nanoparticles by the alkylene group.

The hypotheses issued are verified both by the transport rate and by the efficiency of the silver ion separation process. Both undecenoic acid and undecylenyl alcohol can be used to separate silver ions from equimolar solution with lead ions.

Based on data obtained by varying the main experimental parameters, it can be stated that the ion transport in the membrane system takes place according to a mechanism and kinetics involving the migration of ions from the source phase to the membrane interface, their uptake into the membrane by the carrier, diffusion and convection (magnetically induced) through the membrane, the uptake of the metal ion in the receiving phase from the carrier by interaction and ion exchange with the hydronium ion from the receiving phase. The protonated carrier returns to the source phase due to the pH gradient, but also by the convection provided by the magnetic nanoparticle.

The transport mechanism is widely studied [10,11,21,22], and the intervention of the hydrodynamic parameters improves both the speed and efficiency of the whole process [42,43,65,66].

The kinetics and mechanism of the silver ion transport in the studied system (Figure 2) require in-depth research because in this situation three interconnected dispersed systems are present: the droplets from the source phase through the membrane (in the area of the walls of the permeation module), the nanoparticles transporting from the membrane and the droplets of the receiving phase that cross the membrane (in the central part of the permeator). The surface of the droplets in the source phase or in the receiving phase depends on the flow rate that we impose and must be followed by video means. Although the increase in flow rates favors transport, this must be limited due to the appearance of decanol–aqueous phase emulsions.

## 4. Conclusions

The permeation module with *n*-decanol membrane, undecenoic acid carriers, undecylenyl alcohol and convection promoters of iron oxides/silver and iron oxides magnetic nanoparticles allows the verification of the characteristics of silver and lead ion transport by varying the flow of source and receiving phases, pH adjustment of the receiving phase and stirring regime with magnetic nanoparticles.

Under the conditions of the optimized experimental results (pH = 7 of the source phase, pH = 1 of the receiving phase, the flow rate of 30 mL/min for the source phase and 9 mL/min for the receiving phase, 150 rot/min agitation of magnetic nanoparticles) separation efficiencies of silver ions of over 90% were obtained for the transport of undecenoic acid and about 80% for undecylenyl alcohol.

In the case of the considered carriers, undecylenic acid and 10–undecylenyl alcohol, the use of iron oxide nanoparticles is more effective than the use of silver and iron oxide nanoparticles, most likely due to the effect of the alkylene group.

The separation of silver and lead ions in the studied system leads to separation factors between 6 and 9, under the specified hydrodynamic conditions the most efficient system being *n*–decanol–10–undecylenic acid–iron oxide nanoparticles.

## Data Availability

Not applicable.
